# Surgical site infection and its associated factors following cesarean section in Ethiopia: a cross-sectional study

**DOI:** 10.1186/s13104-019-4325-x

**Published:** 2019-05-27

**Authors:** Getnet Gedefaw Azeze, Asmamaw Demis Bizuneh

**Affiliations:** 1 Department of Midwifery, Faculty of Health Sciences, Woldia University, Woldia, Ethiopia; 2 Department of Nursing, Faculty of Health Sciences, Woldia University, Woldia, Ethiopia

**Keywords:** Surgical site infection, Cesarean section, Ethiopia

## Abstract

**Objective:**

This study aimed to determine the magnitude and associated factors of surgical site infection following cesarean section at Felegehiwot referral hospital, Amhara, Bahir Dar, Ethiopia, 2018. An institution-based retrospective cross-sectional study was conducted from April 1 to May 30, 2018, at Felegehiwot referral hospital. Retrospective chart review was implemented on 383 women who gave birth via cesarean section at Felegehiwot hospital from October 1, 2016 to September 30, 2017. Systematic random sampling technique was implemented to select patient medical charts.

**Results:**

This study revealed that the prevalence of surgical site infection following cesarean section was 7.8% with the [95% CI 5.2–10.5%]. Rupture of membrane before cesarean section (AOR = 13.9, 95% CI 2.99–64.8), vertical skin incision/longitudinal abdominal incision (AOR = 4.77, 95% CI 1.74–13.06), duration of operation lasting longer than 30 min (AOR = 4.9, 95% CI 1.8–13.1), interrupted skin closure technique (AOR = 6.29, 95% CI 2.07–19.11) were statistically associated with surgical site infection following cesarean section.

**Electronic supplementary material:**

The online version of this article (10.1186/s13104-019-4325-x) contains supplementary material, which is available to authorized users.

## Introduction

According to World Health Organization (WHO) cesarean section is the birth of a fetus, placenta & membranes through incisions in the abdominal wall and the uterine wall after 20 weeks of pregnancy [1].

Surgical site infection (SSI) is defined as infections that occur at or near surgical incision within 30 days of operation or after 1 year if an implant is placed. Post cesarean wound infection classified as superficial, deep and organ surgical site infection based on the involved tissues or organs [1, 2].

Surgical site infections (SSI) are the most common postoperative complications which accounts for $3.2 billion in contributable cost per year in hospitals which are giving acute care. Surgical site infections (SSI) are the most common reason to be (20%) unplanned admitted after discharging of the patient to their home [3, 4].

Surgical wound irrigation has an important role to prevent surgical site infection by applying antiseptic solution of chlorhexidine gluconate. Surgical wound irrigation is a recommended strategy to make a better research evidenced based guidelines and improving surveillance system which help the formation of a parameter for health personnel to prevent of infections associated with health care, especially post operative wound infection [5].

Cesarean Section Optimal Antibiotic Prophylaxis (C/SOAP) trial reported using of adjuvant azithromycin prophylaxis decreases’ the incidence of uterine infection, hematometra, postoperative wound infection, and other different types of wound and wound related infections happening within 6 weeks after non elective cesarean delivery having a magnitude of 5.9%. Postpartum use of prophylaxis was reduced by 4.3% [6].

The result of the World Health Organization (WHO) showed that surgical site infections are frequently reported type of hospital-acquired nosocomial infection in both low-and middle-income countries with a reported cumulative incidence of 11.8% [1, 2].

Surgical site infection following cesarean section is not only the most common postoperative complications but a leading predisposing factor for the widespread aversion to cesarean section in less developed and developing countries particularly in African countries.

Even though improvements in surgical techniques, preventive strategies and methods that are designed to reduce post wound infections and availability of prophylactic antibiotics, the magnitude of morbidity and mortality related to surgical infections still remains to be the major nosocomial postoperative complication throughout the world [7].

Post wound infection is one of the most common postoperative complications after cesarean section in both developed and developing countries. Surgical site infection surveillance system is a must to implement before, during and after cesarean section to get a controlled, accurate and standardized magnitude. Any infection of the abdominal wound complicating cesarean section should be decreased through strict preventative strategies [8–10].

The aim of this study helps to strengthen the surveillance system, to give feedback on the proportion of post wound infection after cesarean section and to make alert health practitioners about the prevention of post wound infection in the hospitals. Besides, early prevention of surgical site infection helps reducing individual expenses, hospital costs and decreasing the opportunity of to be infected by other nosocomial infections in the ward.

## Main text

### Methods

This study was implemented at Felegehiwot referral hospital which found in Bahir Dar town, Amhara region, 565 km far from Addis Ababa. According to the Ethiopian central statistical agency report, the total population of the Bahir Dar town administration was 221,991 in 2007. Among these, 108,456 were males, and 113,535 were females [11]. Obstetrics and Gynecology department of Felegehiwot referral hospital has Gynecology ward, high risk, postnatal, labour, OPD, and MCH unit with a total of 82 beds [11]. An institution-based retrospective cross-sectional study was conducted from June 1 to May 30, 2018, among women who had cesarean delivery at Felegehiwot referral hospital from October 1, 2016, to September 30, 2017.

#### Study design

Hospital-based quantitative cross-sectional study design was conducted using a retrospective chart review.

#### Source population

All charts of women who gave birth via cesarean section at Felegehiwot referral hospital.

#### Study population

All selected charts of women who gave birth via cesarean section at Felegehiwot referral hospital from October 1, 2016 to September 30, 2017.

#### Eligibility criteria

##### Inclusion criteria

All cards of women who underwent cesarean delivery during the study period.

##### Exclusion criteria

All women referred from other health care facilities for the diagnosis of SSIs.

Women who had uterine rupture.

#### Sample size determination and sampling procedure

Sample size was determined using a single population proportion formula [n = [(Z_a/2_)^2^ * P (1 − P)]/d^2^] by assuming the single population proportion formula with a 95% confidence level of Z a/2 = 1.96, marginal of error 5%, and proportion of surgical site infection of 6.8% at Maichew, General hospital. Overall the minimum sample size based on single population proportion formula was 334. Since the study was cross section we used also double population proportion formula so as to get appropriate sample size using Epi info version 7.2 stat calc programs, then the sample size was determined 383.

#### Sampling procedure

A total of 1915 women who have given birth via cesarean section were recognized over the last 1 year at Felegehiwot referral hospital from October 1, 2016 to September 30, 2017. From them using systematic sampling technique in every four intervals 383 patient cards were identified and traced using registration number. From a total of 1624 cesarean section procedure, 383 sample sizes was taken by systematic random sampling technique method. Out of 1915 women underwent cesarean section, 291 women were not eligible for our study.
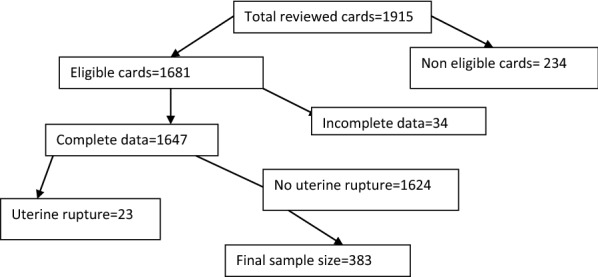


#### Data collection procedures

Information on socio-demographic factors, obstetrics related factors, operation and anesthesia related factors was collected by using checklist. Midwives who have diploma qualification were recruited, trained and collected the data. Overall, the data collection process was strictly supervised by both the principal investigator and supervisors. To assure and control the quality of data, validated tool, pretest double data entry and cross checked system were implemented.

#### Data analysis

The collected data were coded, entered using EPI data version 4.2 and analyzed via SPSS version 23 respectively. Frequencies and summaries were presented in different forms of descriptive statistics by using tables, graphs and figures. Those variables having p ≤ 0.2 in the bivariate analysis were considered as a candidate for multivariable logistic regression analysis. Multi-collinearity was evaluated to distinguish linear correlation among the independent variables using standard errors and variance inflation factors. The degree of association between dependent and independent factors was evaluated by adjusted odds ratio with 95% CI with p value < 0.05. Finally, level of statistical significance was declared at p-value < 0.05.

### Results

#### Socio-demographic characteristics of women following cesarean section

Of the total 383 women who had cesarean section included in the analysis, 238 (62.1%) were from urban residence. The age of women were ranged 18–43 years with a mean and standard deviation of 28.1 ± 5.7. The great majority of 375 (97.9%) women who had cesarean section were married (Table 1).

**Table 1:** Socio-demographic characteristics

Characteristics	Frequency	Percent
Age		
≤ 19	16	4.2
20–34	305	79.6
≥ 35	62	16.2
Religion		
Orthodox	316	82.5
Muslim	58	15.1
Protestant	9	2.4
Residence		
Urban	238	62.1
Rural	145	32.9
Marital status		
Married	375	97.9
Others^a^	9	2.1

Others^a^ (single, divorced and died)

#### Medical related factors

Among 383 women who underwent cesarean section, 40 (10.4%) women had preeclampsia/eclampsia and 17 (4.4%) women who had overt or preexisting diabetes mellitus (Additional file 1: Table S1).

#### Obstetric related factors

From the total of 383 women following cesarean section, more than three fourths (80.7%) of them had antenatal care (Additional file 2: Table S2).

#### Operation and anesthesia related factors

All women who underwent cesarean section have taken antibiotics prophylaxis within 30 min before operation. Regarding the type of abdominal incision, 332 (86.7%) was pfannenstiel incision (Additional file 3: Table S3).

#### Magnitude of surgical site infection following cesarean section

This study revealed that the magnitude of post wound infection following cesarean section in this study was 7.8% (5.2–10.5). Among 30 post wound infection women, 23 (76.7%) and 7 (23.3%) of them were developing superficial and deep surgical site infection respectively. Regarding the detection time of the surgical site infection, 22 (73.3%) of surgical site infection were detected after discharge.

#### Indication of cesarean section

**Fig. 1:**
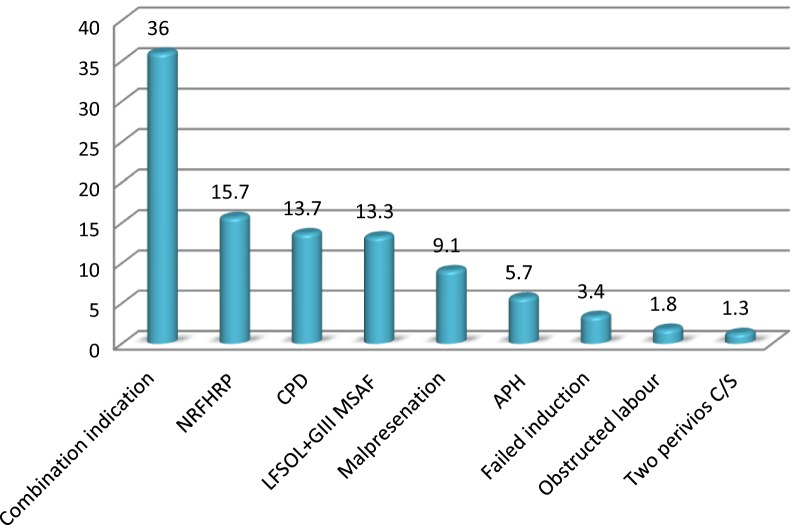
Indication of cesarean section

Out of 383 women who underwent cesarean section non-reassuring fetal heart rate pattern 60 (15.7%) and cephalopelvic disproportion (CPD) 52 (13.7) were the major predisposing factors for cesarean section (Fig. 1).

#### Risk factors associated with surgical site infection following cesarean section

Duration of operation lasting longer than 30 min, rupture of membrane before surgery, longitudinal (vertical) abdominal incision, and interrupted skin closure technique were significantly associated (p < 0.05) in multivariable logistic regression using backward stepwise regression.

Women whose membrane ruptured before cesarean section were 13.9 times more likely risk for surgical site infections than those whose membrane was intact [AOR = 13.9, 95% CI 2.99–64.8].

Women who had a vertical or longitudinal type of abdominal incision were 4.77 more likely risk for surgical site infection than women who had pfannenstiel or transverse abdominal incision [AOR = 4.77, 95% CI 1.74–13.06].

The chance of developing surgical site infection among women whose surgery lasting more than 30 min were 4.9 more times than for women whose surgery lasting within 30 min [AOR = 4.9, 95% CI 1.8–13.1].

The odds of surgical site infections were higher among mothers who had interrupted type of skin closure than who had subcutticular type of skin closure [AOR = 6.29, 95% CI 2.07–19.11] (Table 2).

**Table 2:** Bivariate and multivariable association of surgical site infection

Characteristics	Surgical site infection		COR (95% CI)	AOR (95% CI)
	Yes	No		
Onset of labour				
Induced	5	13	9.32 (2.2–39.1)	0.76 (0.07–8.13)
Spontaneous	21	243	2.09 (0.7–6.26)	0.24 (0.03–2.01)
Not in labour	4	97	1	1
Duration of operation				
≤ 30 min	7	175	1	1
> 30 min	23	178	3.23 (1.35–7.72)	4.9 (1.8–13.1)*
Rupture of membrane				
Yes	28	212	9.31 (2.18–39.7)	13.9 (2.99–64.8)**
No	2	141	1	1
Type of skin incision				
Pfannenstiel	19	313	1	1
Vertical	11	40	4.53 (2.01–10.2)	4.77 (1.74–13.06)*
Skin closure technique				
Interrupted	9	20	7.13 (2.89–17.58)	6.29 (2.07–19.11)**
Subcutticular	21	333	1	1
Circumstance of surgery				
Elective	4	115	1	1
Emergency	26	238	3.14 (1.07–9.21)	1.22 (0.3–4.6)
Number of Vaginal examination				
None	5	120	1	1
1–9	20	210	2.28 (0.83–6.24)	0.94 (0.25–3.55)
10+	5	23	5.2 (1.39–19.48)	2.47 (0.46–13.13)

* p = 0.001, ** p = 0.002

### Discussion

The prevalence of surgical site infection following cesarean section in this study was 7.8% (95% CI 5.2–10.5). This study finding is in line with the study finding at Assela and Tigray [12, 13]. The possible reason might be due to all patients having received preoperative antibiotics.

This finding was higher than the value obtained from Brazil (1.44%), Hungary (3.6%) and Rwanda (4.9%) [3, 14, 15]. This might be due to this study was done where people who had poor socioeconomic capacity. Therefore, having poor economic status is major contributory risk factor for developing hospital acquired nosocomial postoperative infections.

The finding of this study was lower than the value obtained from India (24.2%), Zimbabwe (29%), and Uganda (16.4%) [7, 16, 17]. The possible reason for the discrepancy could be due to there was no post discharge follow up since it was not a cohort study. Therefore, patients may develop post cesarean wound infection and they getting treatment by other health institutions.

Rupture of the membrane prior to surgery was associated with surgical site infection in studies conducted in Nepal [18], Kenya [8], Brazil [14], and Ethiopia [12]. Similarly, in this study rupture of membrane before surgery was an independent predictor for surgical site infection following cesarean section. This may be due to the fact that once the membrane is ruptured no longer protective effect to the cervical canal. Therefore, any bacterial infection can get the chance to ascend through the cervical Os since the sterile and protective membrane was ruptured and lost.

In this study, the duration of cesarean section lasting longer than 60 min was an independent risk factor for surgical site infection. This is similar to the study finding which was done in India [19], Ethiopia [20], and Nigeria [9] showed that prolonged duration of the surgical procedure (lasting longer than 30 min) are usually associated with higher rates of surgical site infections. The possible reason might be due to as the length of operation increased, a tissue exposed to potential bacterial infections is also increased. Hence, the contaminated wound during the procedure is high chance of getting infected after the procedure.

Abdominal midline incision was associated with surgical site infection in the studies done in Ethiopia [12, 20], and Nigeria [9]. Similarly, in this study vertical skin incision is independent risk factors for surgical site infection. This might be due to the fact that the regeneration of the muscular fibers and fibroblast proliferation is fast and cosmetic as well since transverse incision has Langer & Kraissl lines of muscular arrangements which are parallel with the incision.

In this study interrupted skin closure technique was an independent risk factor for surgical site infection. The finding of this study is similar with the study done in Ghana [4], Nepal [18] and UK [6]. This could be due to the fact that less precise wound edge alignment is the major limitation of the interrupted skin closure technique. Therefore, inappropriate edge alignment tissues may increase the chance of getting post cesarean wound infection by hindering the proliferation of connective tissue process.

### Conclusion

It has been revealed that the prevalence of surgical site infection following cesarean section was high. Performing appropriate technique of skin incision and skin closure technique, fastening the operation, early detection and intervention of obstetrics complications may reduce the prevalence of surgical site infection.

## Limitation

This study shares the limitations of cross-sectional studies and hence can’t establish a temporal relationship between surgical site infections and explanatory variables. Since the study is retrospective with chart review, some of the variables are not documented.

## Additional files

**Additional file 1: Table S1.** Medical related characteristics of women following cesarean section at FHRH, Ethiopia, 2018 (n = 383).

**Additional file 2: Table S2.** Obstetrics related factors of women having cesarean section at FHRH, Ethiopia, 2018 (n = 383).

**Additional file 3: Table S3.** Anesthesia and operation related characteristics of women following cesarean section at FHRH, Ethiopia, 2018 (n = 383).

## Data Availability

All related data has been presented within the manuscript. The data set supporting the conclusions of this article is available from the authors on request.

## Abbreviations

ACOG: American College of Obstetrics and Gynecology
CS: cesarean section
CDC: Communicable Disease Control
SSI: surgical site infection
WHO: World Health Organization
FHRH: Felegehiwot referral hospital
