# Supplementary material for: Surgical site infection and its associated factors following cesarean section in Ethiopia: a cross-sectional study
Source: BMC Res Notes. 2019 May 27;12:288. doi: 10.1186/s13104-019-4325-x (PMC6537424; doi:10.1186/s13104-019-4325-x)
Supplement: Supplementary file 1 — Additional file 1: Table S1. Medical related characteristics of women following cesarean section at FHRH, Ethiopia, 2018 (n = 383). [file 13104_2019_4325_MOESM1_ESM.docx]

Table S1: Medical related factors

| **Characteristics** | **Frequency (n=383)** | **Percent (%)** |
| --- | --- | --- |
|  |  |  |
| Urinary tract infection Yes  No | 9  374 | 2.3  97.7 |
| Pneumonia  Yes  No | 5  378 | 1.3  98.7 |
| Preexisting diabetes mellitus  Yes  No | 17  366 | 4.4  95.6 |
| Gestational diabetes mellitus  Yes  No | 12  371 | 3.2  96.8 |
| Pre eclampsia/Eclampsia  Yes  No | 40  343 | 10.4  89.6 |
| Anemia  Yes  No | 13  370 | 3.4  96.6 |
| HIV/AIDS  Yes  No | 16  367 | 4.2  95.8 |
|  |  |  |
