# Supplementary material for: Surgical site infection and its associated factors following cesarean section in Ethiopia: a cross-sectional study
Source: BMC Res Notes. 2019 May 27;12:288. doi: 10.1186/s13104-019-4325-x (PMC6537424; doi:10.1186/s13104-019-4325-x)
Supplement: Supplementary file 2 — Additional file 2: Table S2. Obstetrics related factors of women having cesarean section at FHRH, Ethiopia, 2018 (n = 383). [file 13104_2019_4325_MOESM2_ESM.docx]

Table S2: Obstetric related factors

| **Characteristics** | **Frequency(n=383)** | **Percent (%)** |
| --- | --- | --- |
| Parity  1  2-4  ≥5 | 108  215  60 | 28.2  56.1  15.7 |
| ANC visit  Yes  No | 309  74 | 80.7  19.3 |
| Number of visits  1  2-4  ≥5 | 3  295  85 | 0.8  77  22.2 |
| Onset of labour  Induced  Spontaneous  Not in labour | 18  264  101 | 4.7  68.9  26.4 |
| Duration of labour  ≤24hr  >25hr | 234  48 | 82.9  17.1 |
| Gestational age  Preterm  Term  Post term  Unknown | 81  264  4  34 | 21.1  68.9  1.1  8.9 |
| Number of vaginal examination  None  1-9  ≥10 | 125  230  28 | 32.6  60.1  7.3 |
| Rupture of membrane  Yes  No | 240  143 | 62.7  37.3 |
| Duration of rupture of membrane  <12 hr  ≥12hr | 111  128 | 46.3  53.7 |
| Presence of meconium  Yes  No | 63  177 | 26.3  73.7 |
|  |  |  |
