# Supplementary material for: Surgical site infection and its associated factors following cesarean section in Ethiopia: a cross-sectional study
Source: BMC Res Notes. 2019 May 27;12:288. doi: 10.1186/s13104-019-4325-x (PMC6537424; doi:10.1186/s13104-019-4325-x)
Supplement: Supplementary file 3 — Additional file 3: Table S3. Anesthesia and operation related characteristics of women following cesarean section at FHRH, Ethiopia, 2018 (n = 383). [file 13104_2019_4325_MOESM3_ESM.docx]

Table S3: **Operation and Anesthesia related factors**

| **Characteristics** | **Frequency** | **Percent** |
| --- | --- | --- |
| Preoperative hematocrit  ≤30%  > 30% | 69  314 | 18  82 |
| Circumstance of operation  Elective  Emergency | 119  262 | 31.1  68.9 |
| Type of anesthesia  Spinal anesthesia  General anesthesia | 356  27 | 93  7 |
| Surgeon grade  Senior  Resident 4  Resident 3  Resident 2 | 5  109  243  26 | 1.3  28.5  63.4  6.8 |
| Type of abdominal incision  Pfannenstiel  Vertical | 332  51 | 86.7  13.3 |
| Skin closure technique  Interrupted  Subcutticular | 29  354 | 7.6  92.4 |
| Thickness of subcutaneous tissue  ≤2centimetres  >2centimetres | 325  58 | 84.9  15.1 |
| Postoperative hematocrit  ≤30%  >30% | 102  281 | 26.6  73.4 |
| Intra-operative blood lost  ≤500ml  >500ml | 368  15 | 96.1  3.9 |
| Duration of operation  ≤30 minutes  >30 minutes | 182  201 | 47.5  52.5 |
|  |  |  |
